# A Simple Stabilization Method of Reduced Albumin in Blood and Plasma for the Reduced/Oxidized Albumin Ratio Measurement

**Published:** 2009-09

**Authors:** Kazuyuki Kubota, Akira Nakayama, Kenji Takehana, Asami Kawakami, Naoyuki Yamada, Ei-ichiro Suzuki

**Affiliations:** 1 *Institute of Life Sciences, Ajinomoto Co., Inc., 1-1 Suzuki-Cho, Kawasaki-Ku, Kawasaki-Shi, Japan*; 2 *Pharmaceutical Research Laboratories, Ajinomoto Co., Inc., 1-1 Suzuki-Cho, Kawasaki-Ku, Kawasaki-Shi, Japan*

**Keywords:** reduced albumin, human serum albumin, stabilization method, mercaptalbumin, mass spectrometry

## Abstract

Albumin (Alb) is mixture of reduced and oxidized forms. It is physiologically significant to determine Alb(red)%, which is the proportion of reduced Alb in the sum of Alb. However, reduced Alb in both blood and plasma samples is easily converted to oxidized Alb. Accordingly, the stabilization of Alb in samples is necessary to determine precise Alb(red)% values. Alb stabilization in blood or plasma was achieved by pH control and buffer dilution. At least a 50-fold dilution with 50 mmol/l phosphate buffer (pH 6.0) was required for human plasma. For human blood, a 10-fold dilution with 0.5 mol/l sodium citrate buffer (pH 4.3) was required. To measure Alb(red)%, treated samples were applied to HPLC or LC-ESI-TOFMS. We also developed a “pre-incubation method”, to accelerate the oxidative reaction in plasma by heating at 37°C. Alb(red)% values were maintained around the initial value for 48 h after stabilizing human plasma and 72 h after stabilizing human blood. Accelerating the oxidative reaction in plasma produced large differences in the Alb(red)% values between normal and model disease samples. Precise Alb(red)% values were routinely obtained under the stabilization control. Additionally, pre-incubation of the plasma before measurement is useful to enhance the difference between normal and disease samples.

## INTRODUCTION

Human serum albumin (HSA) is the most abundant protein in plasma (∼40 mg/ml or 0.6 mmol/l), and accounts for 50–60% of total plasma protein (75–80 mg/ml) (1). Albumin (Alb) is synthesized in the liver and secreted into the circulation (2), where it plays many important roles, including maintenance of appropriate osmotic pressure (3), ligand binding and transportation of bilirubin (4), amino acids (5), fatty acids (6), hormones (7), metal ions (8) and various drugs (9), it also acts as a radical scavenger (10), and a source of amino acids during malnutrition (11).

Quantitative determination of albumin is employed in clinical examinations. Alb concentrations are used as an indicator of malnutrition and impaired hepatic function in current clinical laboratory testing (12). Methods using bromocresol green (BCG) and bromocresol purple (BCP) are mainly employed in the quantitative determination of albumin (13, 14). However, both methods have various disadvantages: the BCG method is not specific to albumin and the BCP method is sensitive to the microheterogeneity of Alb, that is, it is affected by the proportion of oxidized albumin. Thus, the development of a more qualitative and quantitative measurement for albumin is required.

Alb is known as a microheterogeneous protein since it can be classified into many types, such as, reduced, oxidized, glycated (15, 16) and a multimeric complex. HSA (66 kDa) is essentially a single-chain polypeptide of 585 residues. All mammalian albumins for which the structure is known have a single thiol group resulting from an unpaired cysteine at position 34. The thiol of Cys34 makes up most of the mercaptan of plasma, in which all of Cys34 is in the SH (mercaptan) form, and is termed reduced albumin (Alb(red)). We have abbreviated human albumin as HSA(red), rat as RSA(red) and mouse as MSA(red) (17, 18). We have also abbreviated, oxidized albumin (Alb(ox)), which is formed from a disulfide bond reversibly oxidized by a compound containing a thiol group such as cystein or glutathione; the human form is abbreviated as HSA(ox), rat as RSA(ox) and mouse as MSA(ox).

We previously reported LC-ESI-TOFMS and FTMS measurements that prove the primary structure of reversible HSA(ox). The structure of the main HSA(ox) consists of a cystein adduct via a disulfide bond at the thiol of Cys34 (11). On the basis of the determined structure of HSA(ox), we prepared both HSA(red) and HSA(ox) standards from human plasma. Using these standards, we examined various properties, including protease susceptibility, ligand-binding and free-radical scavenging activity. The results suggested decreased levels of HSA(red) impairs HSA function in a broad range of pathological conditions. It is therefore important to determine the Alb(red)%, which is the proportion of Alb(red) in total albumin.

Recently, the Alb(red)% measurement has been receiving increasing attention as a discrimination indicator between normal and various disease states. In fact, it has been reported that Alb(red)% tends to be lower in conditions such conditions as liver diseases (19), renal dysfunction (20), diabetes mellitus (21) aging (17, 22), and fatigue (23). Sogami *et al*. first reported the separation of reduced and oxidized HSA using HPLC (18). However, there is a major problem in the measurement of Alb(red)%: Alb(red) in blood or plasma is easily converted into HSA(ox) due to natural oxidation. As a result, Alb(red)% values tend to be lower over time. Thus, it is difficult to obtain precise and reliable Alb(red)% values. Until this problem is solved, samples must be analyzed immediately after blood collection. However, in most clinical examinations there are a great number of samples, making it impossible to measure immediately after blood collection.

In this paper, we report on the development of Alb(red) stabilization after blood collection for Alb(red)% measurement. In addition, we have evaluated the Alb(red)% value using ESI-TOFMS and we have established an Alb(red) stabilization method, which is a combination of pH control and buffer dilution, allowing precise and reliable Alb(red)% value measurements in clinical samples.

The key factors in the stabilization method are pH control and dilution rate. In addition, using solid-phase extraction techniques after stabilization of reduced Alb, we could rapidly determine the Alb(red)% in 5 min per sample by means of flow-injection into ESI-TOFMS.

Furthermore, we developed a “pre-incubation method”, where samples are incubated at 37°C for a constant time, prior to sample stabilization, to accelerate the oxidative reaction. We demonstrated the potential/possible diagnostic utility of the pre-incubation method to plasma samples from rats treated with multiple CCl_4_ administration as a hepatic cirrhosis model and to mouse diabetes model samples. Using the pre-incubation method, the rates of change of Alb(red)% in the diseased samples were larger when compared with normal samples. Although there were no differences between normal and disease model samples, the difference of them became dramatically apparent by applying “pre-incubation method” which are enhanced of oxidation of albumin. Were are able to distinguish between normal and disease model samples by the difference in oxidative rates in pre-incubation method. The “pre-incubation method” has a high availability to the diagnosis of health condition. In the discussion, we evaluated the availability of the pre-incubation method based on our results.

## MATERIALS AND METHODS

### Reagents

Acetonitrile, formic acid, sodium hydrogen carbonate, sodium carbonate, ammonium acetate and sodium sulfate were purchased from Kanto Chemicals Co. (Tokyo, Japan), only acetonitrile was HPLC grade. Citric acid monohydrate, l-cystein, l-cystine dichloride, glutathione reduced form and glutathione oxidized form were purchased from Wako Pure Chemical Industries (Osaka, Japan). dl-homocystein and dl-homocystine were purchased from SIGMA-ALDRICH (Tokyo, Japan).

### Buffers

Buffer A: 50 mmol/l sodium phosphate buffer (pH 6.0); buffer B: buffer A containing 1.5 mol/l potassium chloride; buffer C: 0.1 mol/l sodium carbonate buffer (pH 9.0); buffer D: 0.1 mol/l sodium phosphate buffer (pH 7.3) containing 0.9%[w/v] sodium chloride; Buffer E: 0.5 mol/l sodium citrate buffer (pH 4.3).

### Whole Blood or plasma collection

Approximately 1 ml of whole blood was collected from healthy volunteers or animals using a vacuum tube containing heparin or 0.5 mol/l citrate buffer (pH 4.3). Heparin was added as an anticoagulant, meanwhile citrate was added as anticoagulant and stabilizer for reduced albumin. In accordance with the regulations set by the Human Investigations Committee of the Ajinomoto Cooperation, informed consent was obtained from all healthy volunteers. Each plasma fraction was separated by centrifugation (4°C for 15 min, 2000 g). After collection, plasma was immediately immersed in liquid nitrogen until frozen to maintain the stability of Alb(red): samples were then stored at −80 °C until analysis.

### Stabilization of plasma samples using buffer

Stored plasma was thawed on ice, without shaking, diluted 100-fold with buffer A, and analyzed by LC-ESI-TOFMS, which is HPLC (LC-Packings Famos-Ultimate-Switchos nanoLC system, Amsterdam, Netherlands) coupled to ESI-TOFMS (Bruker Daltonics Inc., Billerica, MA, USA). Two μl of plasma sample was diluted 100 times with buffer A. The diluted sample was filtered using a 0.1 μm cut-off membrane filter tube. Subsequently, 100 μl aliquots of filtered sample were transferred to a sample vial. Two μl of each sample was injected into the pre-column (100 mm × i.d. 200 μm, length packed with monolith C18; GL Science Inc., Tokyo, Japan). Albumin was eluted using a 20 min linear gradient method with 75/25 water/acetonitrile containing 0.1% formic acid (solution A) to 10/90 water/acetonitrile containing 0.1% formic acid (solvent B). All albumin samples were desalted and concentrated using the column switching method. Eluted albumin from the separation column was introduced into ESI-TOFMS (microTOF^®^; Bruker Daltonics Inc.).

### Solid phase extraction and ESI-TOFMS Measurement of plasma samples

Collected blood from healthy volunteers was mixed with heparin or buffer E at a volume ratio 9:1. Samples were then centrifuged to give plasma components. Twenty μl of plasma was added to 1.98 ml of Buffer A. A solid phase extraction (SPE) column (Bond Elute-C18 EWP 200mg/3cc, Varian, Inc., CA) was initialized and equilibrated. Initialization was performed with solvent B (2 ml), equilibration was performed with water (1 ml). The above-mentioned diluted plasma sample was applied to the equilibrated SPE column. The column was washed with 10% acetonitrile (2 ml) containing 0.1% formic acid, and albumin was eluted with solvent B. The elute from SPE was transferred to a sample vial. The sample (1 μl) was introduced into the ESI-TOFMS, using the auto sampler of Ultimate 3000 (Dionex, Idstein, Germany). Solvent B, as a mobile liquid phase, was injected into the mass spectrometer at a flow rate of 15 μl/min.

### Data analysis of Alb(red)% using the ESI-TOFMS spectrum

We used MICROTOF® software to determine Alb(red)% in each sample. The top eight high-intensity albumin ions were used to determine Alb(red)%, which was calculated independently for each identically charged ion using the following formula: Alb(red)% = (peak height of Alb(red))/[(peak height of Alb(red))+(peak height of Alb(ox))] × 100. The average of eight values was considered as the Alb(red)% of the sample.

### HPLC conditions

The stored plasma was diluted 50-fold with buffer A and subjected to HPLC. Shodex-Asahipak ES-502N (7.6 mm i.d. × 100 mm DEAE-form, Shodex, Japan) was used as an albumin separation column. Mobile phase A was 50 mM sodium acetate/400 mmol/l sodium sulfate (pH 4.85), and mobile phase B was ethanol. Flow rate was 1.0 ml/min. Gradient conditions were as follows, mobile phase B% was set to 0-0% (t=0–5 min), 0–5% (t=5–30 min), 5-0% (t=30–35 min), 0-0% (t=35–40 min). Albumin was detected by fluorescence (excitation; 280 nm, emission; 340 nm). Sample injection volume was 20 μl.

## RESULTS

### Intact HSA measurements using LC-ESI-TOFMS

**Figure 1:**
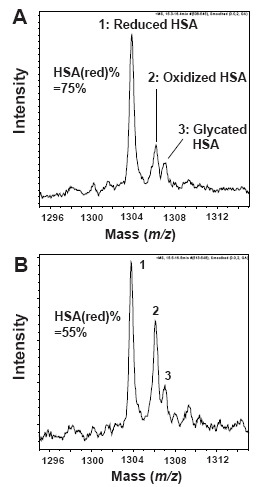
ESI-TOF mass spectra of human serum albumin. All observed peaks in this mass range (*m/z* 1295–1315) were charged [M+51H]^51+^ albumin ions. A, Spectrum of HSA in fresh plasma; B, Spectrum of HSA; incubation for 2 h at 37°C after blood collection. Assigned numbering peak; 1, reduced HSA (HSA(red)); 2, oxidized HSA (HSA(ox):cysteinylated HSA); 3, glycated HSA. HSA(red)% is defined by following formula. HSA(red)% = [(peak 1 height)/(peak 1 height + peak 2 height)] × 100.

Positive multiply-ionized albumin was observed using LC-ESI-TOFMS equipment, with ions distributed from [M+66H]^66+^ to [M+36H]^36+^. We observed structural heterogeneity in HSA and among the structures, we detected HSA(red), HSA(ox) and glycated albumin. Focusing on a mass range of *m/z* 1295–1315, all observed peaks were [M+51H]^51+^ ions (Fig. 1A). In this range, the highest peak intensity was *m/z* 1303.75, which corresponded to intact albumin at 66440.3 mass. This value was close to the theoretical mass of 66437.2 mass, which is calculated from the primary amino acid sequence of HSA, after subtracting 34 Da due to 17 pairs of disulfide bonds. Therefore, we regarded peak 1 as HSA(red) which is a non-posttranslational modification. After allowing natural oxidation to occur by incubating the human plasma samples at 37°C for 2 h, the intensity of peak 2 increased (Fig. 1B), so we regarded peak 2 as HSA(ox). The molecular weight of HSA(ox) was 119.8 mass larger than HSA(red), suggesting the main structure of HSA(ox) included a Cys adduct attached to HSA via a disulfide bond. The heavier peak 3 at *m/z* 1306.90, which correlated to 162.9 mass heavier than reduced HSA, was suggested to be glycated HSA.

Furthermore, the HSA(red)% values in fresh and incubated plasma were 75% and 55%, respectively, suggesting reduced HSA is unstable in plasma. If no treatments were performed, the oxidative reaction progressed, and reduced Alb in fresh plasma was converted into oxidized Alb.

### The stabilization of reduced Alb in plasma

**Figure 2:**
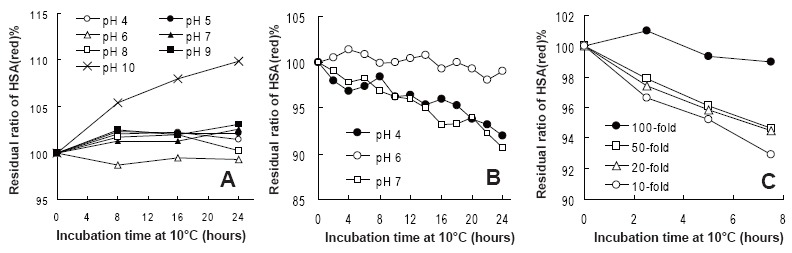
The residual ratio of Alb(red)% change over time at various pH values and dilution rates. A, The residual ratio of HSA(red)% at various pH values (pH 4 to 10); B, the residual ratio of RSA(red)% at pH 4, 6 and 7; C, Each dilution rate of the residual ratio of RSA(red)% from 10 to 100-fold.

Stabilizing Alb(red) in plasma samples is the key factor in measuring Alb(red)% accurately. We optimized the pH control and dilution rate in both human and rat plasma. First, we conducted pH control using various buffers between pH 3 and 10. The HSA(red)% values in human plasma were maintained close to the initial value at pH 4.0 to pH 9.0 (Fig. 2A). The temperature on the sample vial tray was approximately 10°C. At pH 10, however, a tendency towards an increase in the HSA(red)% values over time was confirmed. At pH 3.0, the signal derived from Alb ions was not observed by mass spectrometry. Furthermore, extending the incubation time to 48 h did not affect the HSA(red)% values between pH 4.0 and pH 7.0 (data not shown).

When 50 mmol/l phosphate buffer (pH 6.0) was used as the dilution buffer in rat samples on the sample tray, RSA(red)% was hardly changed from the initial value (Fig. 2B).

The dilution rates to stabilize reduced Alb in plasma were more than 100-fold in RSA(red)% (Fig. 2C) and 50-fold in HSA(red)% (data not shown), suggesting reduced HSA in plasma is more stable than reduced RSA in plasma.

### Stabilization of reduced Alb in whole blood samples for clinical examination

The data so far relates to the stability of the plasma fraction. The stability of reduced HSA in whole blood was also investigated, since this is more important in clinical examinations.

Table 1 shows changes in the residual ratio of Alb(red)% at 4°C over time, obtained by LC-ESI-TOFMS and HPLC measurements. In heparin-added samples, the residual ratio of Alb(red)% decreased to 92.0–93.5% for 48 h at 4°C. Alternatively, after addition of 0.5 mol/l citrate buffer (pH 4.3) to 1/9 volume of the blood sample, the residual ratio of Alb(red)% tended to maintain its initial value (100–101%) for 48 h at 4°C. These results clearly demonstrate that Alb(red) in whole blood remained stable for 2 days in a refrigerator after addition of 0.5 mol/l citrate buffer (pH 4.3). Similar results were obtained using HPLC. The residual ratio of Alb(red)% after 72 hour incubation in a refrigerator was 99.4%-100.4%, and fluctuations were not observed.

**Table 1:** The stability of HSA(red)% in whole blood samples

Volunteer#	Measurement Method	Anticoagulant^a^	Store time^b^ / Residual ratio of HSA(red)%			
			0 h	24 h	48 h	72 h

1	LC-ESI-TOFMS	Heparin	100	97.8	93.5	-
2			100	97.2	92.1	-
3			100	95.4	92.0	-
1		Citrate^c^	100	100.7	100.2	-
2			100	100.3	100.7	-
3			100	99.9	100.1	-
4	HPLC	Citrate^c^	100	100.6	-	100.0
5			100	10.8	-	100.4
6			100	100.4	-	99.7
7			100	100.3	-	99.4
8			100	100.4	-	100.1

a Each additive was applied to the whole blood sample;

b Samples were stored in the refrigerator (at 4°C);

c Citrate, which consists of 0.5 mol/l citrate buffer (pH 4.3), was added to the blood at 1/9 the volume of the collected blood.

### The rapid measurement of Alb(red)% by SPE treatment and flow-injection analysis

Samples containing Alb were desalted using a Bond Elute-C18 EWP column, which was utilized as reversed-phase SPE treatment. Using the desalting step meant we were able to introduce the sample directly into the ESI-TOFMS. The combination of SPE treatment and flow-injection analysis shortened the Alb(red)% measurement time to 5 min per sample. This approach results in rapid analysis of HSA(red)% values.

### Reproducibility of Alb(red)% measurements by LC-ESI-TOFMS

We evaluated the reproducibility of Alb(red)% measurements in both rat and human plasma by a combination of citrate buffer addition and SPE treatment. RSA(red)% had high reproducibility values, CV% was below 2.6% (intra-day, n=4) and 1.35% (inter-day, n=4), and HSA(red)% also had high reproducibility values, CV% was below 0.5% (intra-day, n=4) and 1.55% (inter-day, n=4) (Table.2).

**Table 2:** Measurement of Alb(red)% stability using LC-ESI-TOFMS

Species^a^	Analysis Day	1st round	2nd round	3rd round	Mean ± SD (intra-day)	CV%	Mean ± SD (inter-day)

Rat	Day 1	66.3	65.6	66.4	66.1 ± 0.4	0.70%	66.6 ± 0.9 (CV%:1.35)
	Day 2	66.0	65.6	66.2	65.9 ± 0.3	0.50%	
	Day 3	68.6	67.8	67.3	67.9 ± 0.7	1.03%	
	Day 4	63.3	66.7	65.8	66.5 ± 1.8	2.60%	
Human	Day 1	77.4	77.9	77.4	77.6 ± 0.3	0.37	77.9 ± 1.2 (CV%:1.55)
	Day 2	77.1	77.7	77.4	77.4 ± 0.3	0.39	
	Day 3	79.6	79.9	79.4	79.6 ± 0.3	0.32	
	Day 4	77.0	77.2	76.5	76.9 ± 0.4	0.47	

a All samples are normal samples or from healthy volunteers.

### Availability of the pre-incubation method

Our results implied that preliminary incubation of samples at 37°C caused a decrease in Alb(red)% values due to acceleration of natural oxidation. We termed this treatment “pre-incubation method”, and investigated whether a difference would arise between species and health conditions in the Alb(red)% change rate.

**Figure 3:**
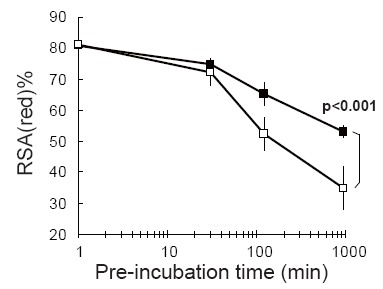
The comparison of the transitional change of RSA(red)% values after pre-incubation treatment. (black box); Normal rats, (white box); CCl_4_ administrated rats, the liver cirrhosis model (for each group N=4). RSA(red)% values were determined using LC-ESI-TOFMS. Each of the error bar was shown as standard deviation (N=4).

**Figure 4:**
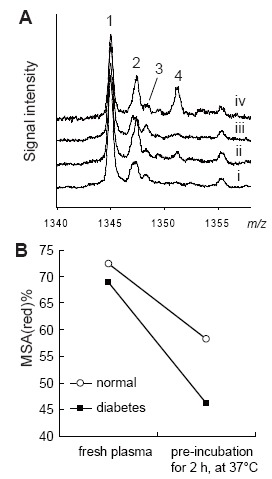
Changes in the profile of mouse serum albumin, with or without pre-incubation. A, LC-ESI-TOFMS spectra of mouse serum albumin (MSA: *m/z* 1340–1358). In this *m/z* range, all observed Alb ions were charged [M+50H]^50+^ MSA ions. Line i is normal mouse plasma without pre-incubation. Line ii is normal mouse plasma with pre-incubation for 2 hour at, at 37°C). Line iii is KK-Ay (diabetes model) mouse plasma without pre-incubation. Line iv is KK-Ay mouse plasma with pre-incubation for 2 hour at 37°C. 1, reduced form MSA; 2, Cys addition MSA, 3, glycated MSA; 4, glutathione addition MSA; B, The shift in MSA(red)% values with or without preincubation (for 2 h, at 37°C).

In the first experiment using the pre-incubation method, we investigated fluctuations in RSA(red)% between normal rats and rats administered multiple CCl_4_ as a hepatic cirrhosis model. Although there were no significant differences before pre-incubation (time at 0 min), there were clear significant differences between the two groups at 120 min pre-incubation (p<0.05) (Fig. 3). Similarly, we applied the pre-incubation method to diabetic model mice. We measured MSA(red)% in both normal and the diabetes model mouse. Without pre-incubation, MSA(red)% in the normal mouse and the diabetes model mouse were 72.4% and 68.9%, respectively. However, pre-incubation drastically increased two kinds of oxidized MSA on the mass spectrum of the diabetes mouse model (Fig. 4A). Based on mass differences between reduced MSA, we speculated that one of the oxidized MSAs was the cystein adducted type and the other was suggested to be the glutathione (GS) adduct form. In particular, the GS adduct form seems to be the discriminative form in the diabetes model. In addition, the proportion of glycated Alb in the sum of albumin was determined using LC-ESI-TOFMS measurements in the same way. The proportions of glycated Alb were as follows: normal sample without pre-incubation (9.9%), normal sample with pre-incubation (9.8%), diabetes model sample without pre-incubation (13.9%), and diabetes model sample with pre-incubation (14.0%). Although, there were no differences between with and without pre-incubation samples, there was a clear difference between normal and diabetes model samples.

## DISCUSSION

Albumin could potentially be used as an indicator of malnutrition and impaired hepatic function. Of particular interest is the proportion of reduced albumin in total albumin. Historically, both HPLC and ESI-TOFMS system have been employed for the measurement of Alb(red)%. Using ESI-TOFMS not only enables the detection of albumin's microheterogeneity, that is, molecular mass information on reduced type, oxidized type, glycated type and multimeric complex, but the Alb(red)% value can also be simultaneously determined. However, since the Alb(red)% in blood and plasma samples are known to fluctuate, due to natural oxidation, sample stability has been a key point in determining reliable Alb(red)% values.

In order to obtain reliable Alb(red)% values from blood collection to sample measurement, our approach was to control pH and dilute samples with buffer. First, we investigated Alb(red)% stabilization by pH control. Albumin's sole SH-group at Cys34 appears to be comparatively more acidic with a pK between 4 and 8 (1). Fig. 2A shows that the HSA(red)% value remains stable between pH 4.0 to pH 9.0. Under alkaline conditions, deprotonation of the SH-group or dissociation of the disulfide bond can occur, producing the Alb thiolate anion as an intermediate in the formation of albumin-SS-compound. As a result of this reaction, the HSA(red)% value fluctuates. However, the RSA(red)% value is not stable even at pH 4.0 or 7.0. Accordingly, it was demonstrated that RSA was less stable than HSA.

Subsequently, we investigated the dilution rate to stabilize reduced Alb in plasma. A higher dilution rate may reduce contact opportunity between albumin and low molecular weight compounds in plasma. Since oxidized Alb consists of a Cys and reduced Alb, the higher dilution rate was more effective in stabilizing reduced Alb in plasma. The HSA(red)% value was maintained close to the initial value after a minimum of a 50-fold dilution of the human plasma sample, even when the sample was left on the sample tray (10°C). We have examined the effect of removing low molecular weight compounds from the plasma sample using affinity columns in Alb preparation. The column can separate albumin and low molecular weight compounds in blood or plasma samples. After removal of low molecular weight compounds, reduced Alb was prevented from converting to oxidized Alb and the Alb(red)% value was maintained close to its initial value after column treatment (data not shown). Thus, it is suggested that low molecular compounds can contribute to the conversion of Alb(red) to Alb(ox).

Stabilizing treatments for reduced Alb can be used in both plasma fractions and whole blood. This is particularly important in the clinical examination room, since most whole blood samples are left at room temperatures for a while before they can be processed. If a stabilizing method could be established, Alb(red)% measurements might become acceptable in the clinical field. Addition of 0.5 mol/l citrate buffer to blood samples stabilized reduced HSA in whole blood for 72 h. This means that all samples can be reliably stored until Alb(red)% measurements, up to 72 h, and transport time from the blood collection room to the specialized agency for HSA(red)% measurement is not a major issue.

In general, heparin is used as one of the typical anticoagulants. Although the plasma sample could be stored for a long period at −80°C, in the whole blood sample, it was difficult to determine the proportion of reduce albumin at −80°C due to hemolytic action. Since the effect of citrate buffer addition to the whole blood is the prevention of hemolysis, the blood sampling tubes including citrate have became commercially available. Therefore, we think the addition citrate buffer has little or no influence on other biochemical examinations of blood. Table 1 shows that heparin as one of the typical anticoagulant have not effect as stabilizer for reduced albumin. Meanwhile, citrate has not only anticoagulant function but also stabilized function for reduced albumin.

Furthermore, by a combination of citrate buffer addition and SPE, we determined HSA(red)% in only 5 min using flow-injection into ESI-TOFMS. Therefore, flow-injection analysis is a rapid measurement, which can accommodate many samples.

Additionally, this method may have applicability to determine not only the Alb(red)% value but the amount of Alb. In Japan, the amount of Alb has generally been determined using either bromocresol green (BCG) or bromocresol purple (BCP) methods. The BCG method lacks HSA specificity and the BCP method is affected by the oxidized Alb percentage in a sample (12). In view of this, our method is a more reliable measurement of Alb and could replace the two previous methods.

We have also devised a “pre-incubation method”. This method purposely utilizes an instability feature of albumin, that is, each reduced albumin in plasma was artificially converted into oxidized albumin at an accelerated pace during a 37°C incubation period. Results showed we could clearly distinguish between normal samples and diseased model samples. Fig. 3 demonstrates that there was little difference in Alb(red)% values between normal samples and the CCl_4_ administrated rat samples immediately after blood collection, however, differences between normal and case samples were observed with pre-incubation time.

The similar usefulness of the “pre-incubation method” were obtained using the diabetes mouse model, in which the biggest difference was observed in glutathione adducted MSA. Free glutathione concentration is decreased in several disease states, despite a constant concentration of total glutathione (24). This suggests the pre-incubation method will shift free glutathione in the blood or plasma samples to glutathione addition products and MSA(red)% values will increase with increasing incubation time.

Oxidized Alb and reduced Alb closely reflect the redox state *in vivo*. We think that the proportion of reduced albumin can be one of the markers which reflect the redox state *in vivo*. Previously, we have compared the radical scavenging activity between non-oxidized and highly oxidized HSA samples. As we had expected, a highly oxidized HSA reduced its radical scavenging activity (11). The function is believed to be ascribed to its single exposed thiol group at Cys34. Because albumin accounts for most of the total plasma thiol content (about 80%), it can act as a major antioxidant in plasma where the amounts of antioxidant enzyme are relatively small. Therefore, the pre-incubation method may be applicable to valid for various diseased states, including those already mentioned. We would like to emphasize that the pre-incubation method complements the previously established stabilization method for reduced Alb in blood or plasma.

We have developed the simple stabilization method for reduced albumin. By using this method, precise Alb(red)% values were routinely obtained under the stabilization control. Additionally, by applying the pre-incubation method, we could clarify the difference between normal and diseased sample.
